# Application of a computational model of natural deep eutectic solvents utilizing the COSMO-RS approach for screening of solvents with high solubility of rutin

**DOI:** 10.1007/s00894-018-3700-1

**Published:** 2018-06-27

**Authors:** Tomasz Jeliński, Piotr Cysewski

**Affiliations:** Department of Physical Chemistry, Collegium Medicum, Nicolaus Copernicus University, Kurpińskiego 5, 85-950 Bydgoszcz, Poland

**Keywords:** Rutin, NADES, Deep eutectic, Screening, Solubility, Modeling

## Abstract

The screening of natural deep eutectic solvents (NADES) to identify those with the ability to strongly solvate rutin was conducted using the COSMO-RS methodology. A NADES model was constructed that took into account the possible ionic and neutral forms of its constituents. The distributions of all forms were computed based on the equilibrium constants of neutralization reactions between amino and carboxylic acids. The proposed model was validated against the experimental solubilities of 15 NADES. A linear relationship between these data and the estimated activity coefficient values was found. The screening encompassed 126 different NADES. It was found that ten of them outperformed the best reference system. The most effective two-component solvent comprised proline combined with 2,3-diaminosuccinic acid, and the solubility of rutin in this solvent was found to be 130% greater than its solubility in the best reference system. The amino acids associated with the highest rutin solubilities were all cyclic, and the use of carboxylic acids with two carboxyl groups and a main chain consisting of two methylene groups with two amino substituents was observed to yield the best rutin solubilities. Because of the acidic properties of rutin, the presence of basic sites on the components of the NADES generally leads to enhanced solubility.

**Electronic supplementary material:**

The online version of this article (10.1007/s00894-018-3700-1) contains supplementary material, which is available to authorized users.

## Introduction

Plants and other living organisms are key sources of pharmacologically active substances, and it is necessary to use a range of extraction techniques to obtain these compounds from organisms. Traditional extraction techniques include a variety of solvents, such as water, ethanol, and other organic solvents, as well as mixtures of them. However, these extraction media have significant disadvantages, including low selectivity and the presence of residual solvent in the sample [1, 2]. Additionally, many compounds exhibit poor solubility in water and other organic solvents, which makes it difficult to extract them [3, 4]. Also, the high volatility and toxicity of many solvents limits their use in the food and pharmaceutical industries. Therefore, there is a need for alternatives that offer enhanced extraction abilities and are more environmentally friendly than traditional solvent extraction.

**Fig. 1:**
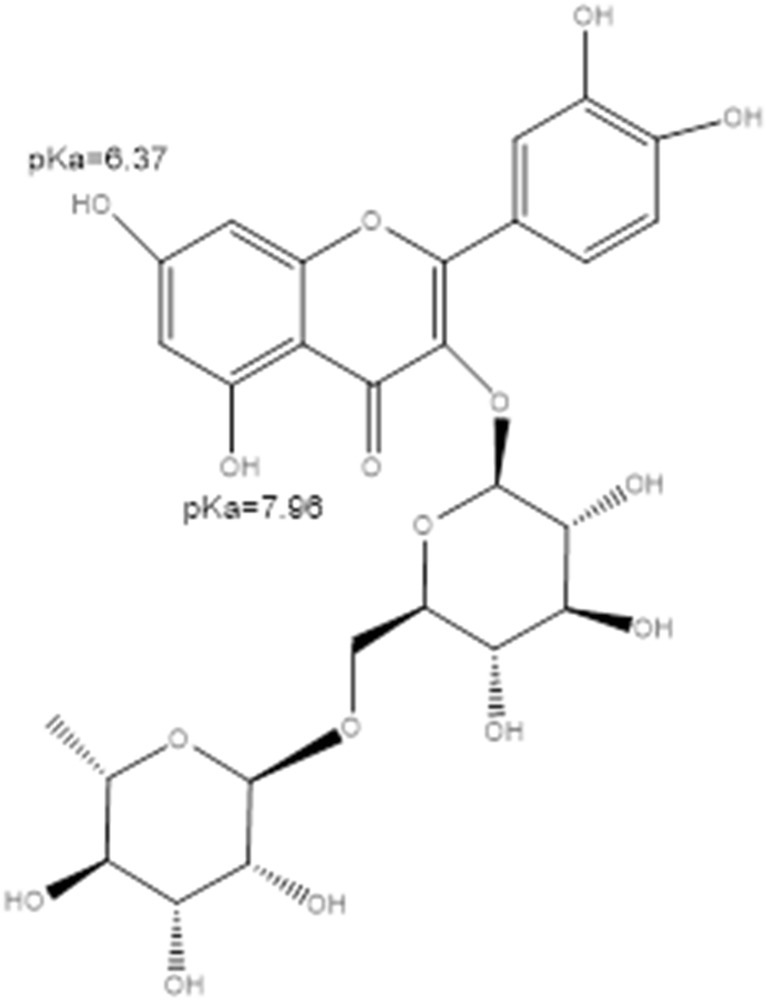
Schematic representation of the structure of rutin, with annotated microacidities of the most acidic sites (estimated via ChemAxon [10])

Rutin (Fig. 1) belongs to class of active substances—the flavonoids—that are obtained from natural sources. The flavonoids are a group of secondary plant metabolites with antioxidant and antimicrobial properties [5]. Rutin, a glycoside of quercetin, can be found naturally in plants and fruits such as potatoes, tomatoes, onions, and other vegetables, as well as in tea. It has attracted the attention of researchers because of its significant antioxidant properties [6], skin protection potential [7], and other health-promoting effects [8]. However, it can be difficult to prepare effective rutin formulations, as it exhibits poor solubility in water [9] and its relatively high solubility in some organic solvents such as ethanol, dichloromethane, and DMSO cannot be fully utilized in pharmaceutical formulations.

Natural deep eutectic solvents (NADES) are a group of bioderived deep eutectic solvents which are composed of two or more compounds that are generally plant-based primary metabolites, i.e., organic acids, sugars, alcohols, amines, and amino acids [11, 12]. On the other hand, deep eutectic solvents can in general be defined as mixtures of solid compounds that form liquids, as these mixtures have melting points below room temperature [13]. This reduction in melting point permits the generation of liquid eutectic solvents, whereby electrostatic or hydrogen-bond interactions between a hydrogen-bond state of the deep donor and an anion are more energetically favorable than the lattice energies of the components of the mixture [14, 15]. NADES possess unique physicochemical properties that distinguish them from and make them superior to regular solvents, especially from economic and environmental perspectives. These properties include low volatility, existence in a liquid state even at subzero temperatures, high potential to extract and stabilize different compounds, biodegradability, sustainability, low cost, and simplicity of preparation [12, 16–19]. Due to the food-grade properties of the components of NADES, extracts obtained using such systems can be consumed by humans without the need for further purification procedures [20]. These properties have led to NADES replacing ionic liquids in many applications, as the usage of ionic liquids in foods and pharmaceuticals is generally avoided nowadays because of their potential toxicity [21, 22]. The field of applications of natural deep eutectic solvents is very broad, and includes for example the dissolution of DNA [11, 12], acting as media for enzyme reactions [23], biotransformations [24], biomass processing [25], the stabilization of pigments [16], and various extractions [26–29]. The pharmaceutical industry is another field in which NADES can be extremely useful; for instance, they can be used to improve the solubility of drugs that are poorly soluble in water [30, 31] and to enhance the bioactivities of dissolved species [32].

Various chemical compounds can form natural deep eutectic solvents, and the properties of such systems can be fine-tuned for specific applications [12, 33]. This abundance of possible formulations makes it impossible to study all of them experimentally. However, in silico methods can offer valuable guidance when attempting to select the most promising systems, as they provide a way to pre-screen systems before performing real-world measurements. Such an approach, which has already been successfully used to study ionic liquids [34], offers important economic and environmental advantages by speeding up the screening procedure and reducing the amounts of chemicals and energy used during the process of selecting the optimal system.

In the present study, the thermodynamic activity coefficient at infinite dilution, as calculated using the COSMO-RS computational methodology, was correlated with experimental values for the solubility of rutin, and the resulting correlation was used to screen for more efficient natural deep eutectic solvents that could be applicable for rutin dissolution. Aside from achieving this practical goal, the present work highlights the importance of the methodological aspects used and permitted the formulation of a reliable NADES model, which is discussed later in this paper.

## Materials and methods

The experimental solubilities of rutin in natural deep eutectic solvents were taken from the work of Faggian and coworkers [35]. Those results, involving fifteen different NADES obtained by combining any of five carboxylic acids with any of four amino acids, were used as a training set for the purpose of validating the computational protocol. During the screening procedure, this initial list was extended such that 21 carboxylic acids and 6 amino acids were mixed in various combinations to give a total of 126 different NADES. This extension of the set of component carboxylic and amino acids that were used to generate the NADES was conducted in a systematic manner based on structural similarities between components. The additional 16 carboxylic acids all contained two carboxyl groups separated by an aliphatic chain augmented with different functional groups, and the pool of amino acids was extended by including two additional cyclic species. The names of all the considered compounds together with their InChI keys are collected in Table 1.

**Table 1:** Names and InChI keys of the compounds used in the study

Code	Name	InChI Key	Code	Name	InChI Key
1	2,3-Diaminosuccinic acid	PGNYNCTUBKSHHL-UHFFFAOYSA-N	15	2,4-Dihydroxyglutamic acid	FTWPXBYNGOWCHI-UHFFFAOYSA-N
2	2,4-Diaminoglutaric acid	LOPLXECQBMXEBQ-UHFFFAOYSA-N	16	Malic acid	BJEPYKJPYRNKOW-UHFFFAOYSA-N
3	4-Amino-3-hydroxyglutamic acid	ROZOJQWPTMSEJM-NUWTVHIESA-N	17	Oxalic acid	MUBZPKHOEPUJKR-UHFFFAOYSA-N
4	Aspartic acid	CKLJMWTZIZZHCS-UHFFFAOYSA-N	18	Malonic acid	OFOBLEOULBTSOW-UHFFFAOYSA-N
5	3-Hydroxyaspartic acid	YYLQUHNPNCGKJQ-UHFFFAOYSA-N	19	Citric acid	KRKNYBCHXYNGOX-UHFFFAOYSA-N
6	3-Aminoglutamic acid	BBJIPMIXTXKYLZ-UHFFFAOYSA-N	20	Succinic acid	KDYFGRWQOYBRFD-UHFFFAOYSA-N
7	Glutamic acid	WHUUTDBJXJRKMK-UHFFFAOYSA-N	21	Tartaric acid	FEWJPZIEWOKRBE-UHFFFAOYSA-N
8	2-Amino-3-hydroxyglutamic acid	LKZIEAUIOCGXBY-UHFFFAOYSA-N	A	Proline	ONIBWKKTOPOVIA-BYPYZUCNSA-N
9	Aminomalonic acid	JINBYESILADKFW-UHFFFAOYSA-N	B	Cycloleucine	NILQLFBWTXNUOE-UHFFFAOYSA-N
10	2-Amino-4-hydroxyglutamic acid	HBDWQSHEVMSFGY-STHAYSLISA-N	C	4-Hydroxyproline	PMMYEEVYMWASQN-UHFFFAOYSA-N
11	2-Hydroxyglutamic acid	HWXBTNAVRSUOJR-VKHMYHEASA-N	D	Ornithine	AHLPHDHHMVZTML-BYPYZUCNSA-N
12	Hydroxymalonic acid	ROBFUDYVXSDBQM-UHFFFAOYSA-N	E	Arginine	ODKSFYDXXFIFQN-BYPYZUCNSA-N
13	3-Hydroxyglutamic acid	ZQHYXNSQOIDNTL-UHFFFAOYSA-N	F	Citrulline	RHGKLRLOHDJJDR-BYPYZUCNSA-N
14	Glutaric acid	JFCQEDHGNNZCLN-UHFFFAOYSA-N			

This includes compounds that were used to validate the theoretical model, as well as compounds selected because they were thought to have the potential to solvate rutin well

The COSMO-RS (Conductor-like Screening Model for Real Solvents) [36, 37] approach can be used to compute the values of the chemical potentials of molecules in liquid solutions. These data enable the estimation of other thermodynamic parameters, such as solubilities or activity coefficients. This approach is recognized to be a valuable and promising theoretical method as it has been successfully used to examine the interactions of organic molecules with ionic liquids [38, 39], water–ionic liquid systems [40, 41], and deep eutectic solvents [42, 43], including in one of our previous studies [34]. Unfortunately, predictions of the absolute values of such thermodynamic parameters employed as activity coefficients have often been found to be poorly accurate and to correlate poorly with the results of experimental measurements. Despite this fact, qualitative trends and correlations can still be very informative about the studied systems, provided that the calibration and validation of the computed values are done against experimental data.

The principles of the COSMO-RS methodology can be found elsewhere [36, 37], so only a brief review is offered here. The COSMO-RS calculation begins by creating a discrete surface around each molecule which is immersed in an ideal virtual conductor. Each of the created surface segments can be characterized by its area and its screening density charge. Three contributions play a role in calculating the total interaction energy between species: hydrogen bonding, the electrostatic misfit, and van der Waals interactions. The screening charge density distribution on the surface of each molecule was converted into a surface composition function called the sigma profile (σ-profile). The σ-profile of the whole system* p*_S_(σ) can be then described as the sum of the σ-profiles of its individual components weighted by their molar fractions. The chemical potential of a surface segment is given by the following equation:

1 $$ {\mu}_{\mathrm{S}}\left(\sigma \right)=-\frac{RT}{a_{\mathrm{eff}}}\ln \left[\int {p}_{\mathrm{S}}\left({\sigma}^{\prime}\right)\exp \left(\frac{a_{\mathrm{eff}}}{RT}\left({\mu}_{\mathrm{S}}\left({\sigma}^{\prime}\right)-e\left(\sigma, {\sigma}^{\prime}\right)\right)\right)d{\sigma}^{\prime}\right], $$

where* e*(*σ*,*σ*′) = (*E*_VdW_(*σ*,*σ*′) +* E*_HB_(*σ*,*σ*′) + *E*_MF_(*σ*,*σ*′)/*a*_eff_). The calculated chemical potentials provide the basis to compute other thermodynamic properties, such as the activity coefficient* γ*, which can be determined as follows:

2 $$ \ln \left({\gamma}_j\right)=\left({\mu}_j^{\mathrm{S}}-{\mu}_j^{\mathrm{P}}\right)/ RT, $$

where* μ*_*j*_^P^ is the chemical potential of the pure compound *j* and* μ*_*j*_^S^ is the chemical potential of compound *j* at infinite dilution in the solvent. For ionic species *j*, the reference state for the activity coefficient is not the pure compound* μ*_*j*_^P^ but infinite dilution of the ionic species in the solvent.

The equilibrium constant of a reaction can be computed based on the Gibbs free energy of reaction:

3 $$ K=\exp \left(-{\Delta  G}_{\mathrm{r}}/ RT\right), $$

where ∆*G*_r_ is defined as the difference in free energy between the product compounds and the reactant compounds.

The molecular structures of the considered compounds were downloaded from the PubChem database [44] in the form of SDF files, and a full conformational analysis was conducted using the COSMOConfX16 software [45]. The conformational analysis was performed at the BP-TZVPD-FINE level both in the gas phase and in water solution. The maximum number of energetically favorable conformers was restricted to ten. This step included a full geometry optimization of each conformer using the BP functional with the def2-TZVP basis set, followed by single point computations using the def2-TZPVD basis set, as available in Turbomole ver. 7.0 [46]. These computations yielded sigma profiles that were used to model the liquid state along with the precise energetics in the gas phase. The final thermodynamic properties were computed using the BP_TZVPD_FINE_C30_1701 parametrization set available in the COSMOthermX software [47]. This level of computation is considered to be the current state of the art for estimating thermodynamic properties based on first-principles computations.

## Results and discussion

The screening of NADES to find the most effective NADES for solvating rutin encompassed a two-step procedure. Initially, based on data collected using the training set, theoretical models were constructed and validated. Their accuracies were assessed by estimating the correlation between computed thermodynamic quantities and measured solubility data. Since the composition of the optimal NADES was unclear, two different types of models were proposed. In the first, NADES solutions were created by mixing together the neutral, undissociated forms of the compounds considered here in unimolar ratios. The second, more realistic, model of NADES took into account the possible dissociation of each species and involved precisely computing their molar fractions based on the values of their equilibrium constants. The performance of each model was checked against a set of experimental data. After ensuring acceptable accuracy, the final step was undertaken, and a comprehensive screening process for the optimal NADES for solvating rutin was performed.

### Construction of the model and validation

**Fig. 2:**
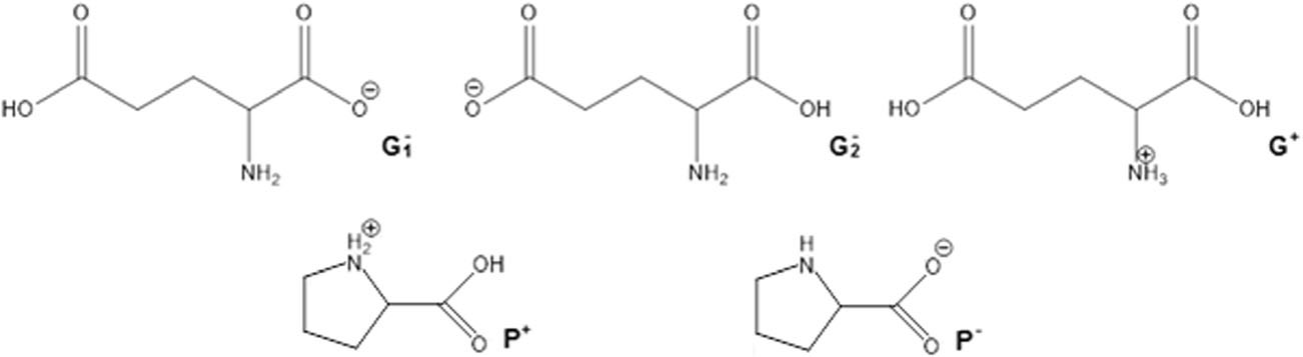
Dominant ionic forms of glutamic acid (*G*) and proline (*P*) considered during the construction of the theoretical model of a NADES comprising these two compounds

**Fig. 3:**
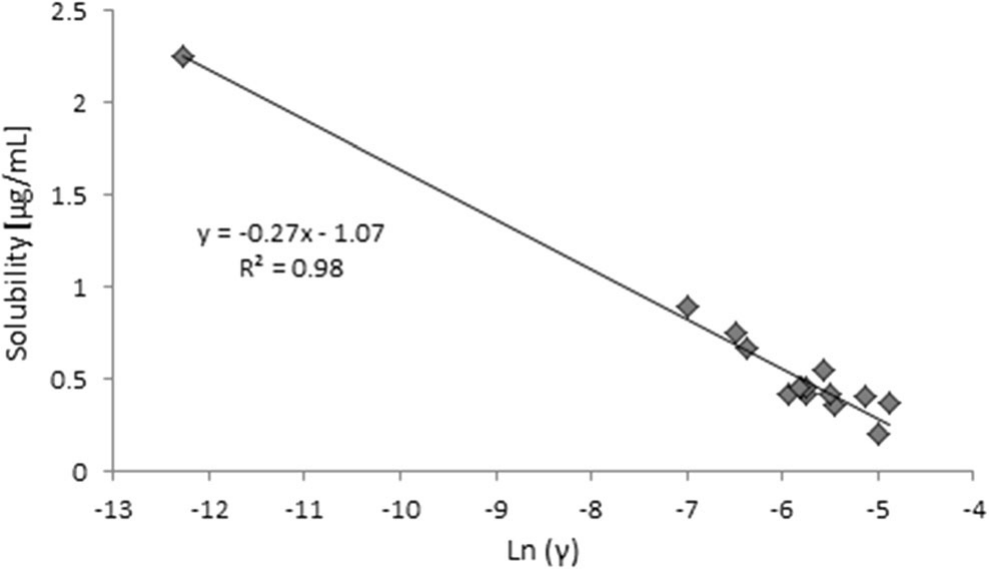
The correlation of the computed activity coefficients at infinite dilution with the solubility of rutin in different natural deep eutectic solvents

In principle, the quantitative compositions of NADES can be obtained from spectroscopic measurements. Unfortunately, such data are usually unavailable, and the resulting uncertainties make it difficult to construct a NADES model. In a reliable theoretical model of a multicomponent solvent, it is necessary to not only include the proper forms of the components but also their concentrations. The simplest model can be formulated by including only neutral, undissociated forms of the studied NADES constituents. This simplified approach is worth considering initially since it is relatively computationally inexpensive. Hence, the construction of the systems studied experimentally and encompassing 15 different natural deep eutectic solvents created from a pool of five carboxylic acids (glutamic acid, malic acid, tartaric acid, oxalic acid, citric acid) and four amino acids (proline, ornithine, arginine, citrulline) is quite straightforward. A unimolar ratio of the constituents was adopted to mimic the regime applied in experimental measurements. Computation of the activity coefficients at infinite dilution according to this simple model led to very poor correlation with the experimental solubility of rutin in NADES, since the correlation coefficient* R*^2^ was equal to 0.167. It was clear, then, that a more sophisticated and realistic model had to be formulated. To achieve a more accurate representation of NADES components, three steps were undertaken. The initial one involved finding the possible ionic forms of the constituents of the deep eutectic solvent that could be present in the considered system. The number of expected forms could be quite large considering that several isomeric forms could occur. Thus, all of the potential structures of every species involved in each NADES had to be carefully identified. Figure 2 shows an example of such an analysis for the proline–glutamic acid system. Glutamic acid dissociation can lead to two distinct anions and one cation, while the dissociation of proline can yield just one cation and one anion. Pinning down the actual formulation of this NADES model, however, required an analysis of the population distribution of all seven forms in solution (i.e., two neutral and five ionic forms). This was achieved by considering the three equilibria presented in Table 2 for the example system. The values of the calculated equilibrium constants allow the actual composition of all ionic and neutral species to be identified. Since all of the reactions are generally mutually dependent, there is no analytic solution, and a numerical procedure had employed to compute unknown concentrations. This is a typical problem encountered when studying chemical reactions, and one that is routinely solved in chemical engineering. This general procedure was adopted to characterize all of the NADES used during the validation stage, and the results of this analysis afforded information on the final composition of each of NADES considered in this work. In Table 3, the dominant forms of the species involved in the example proline–glutamic acid system and all other natural deep eutectic solvents used to validate the model are shown together with their molar fractions in the final mixtures. It is also worth mentioning that these compositions can be used to not only compute the solubility of rutin but also to model other theoretical problems involving these NADES. Here, the modeled solvents were used to calculate the activity coefficients of rutin at infinite dilution. Interestingly, the calculated values correlate quite well with the experimentally obtained solubility of rutin, as shown in Fig. 3.

**Table 2:** Types of reactions taken into account when creating a model of a NADES comprising glutamic acid (G) and proline (P)

Reaction	Equilibrium	*K*	Δ*G*° (kcal/mol)
K_1_	G + P = G_1_^−^ + P^+^	1.62 × 10^5^	−7.11
K_2_	G + P = G_2_^−^ + P^+^	1.02 × 10^4^	−5.48
K_3_	G + P = G^+^ + P^−^	6.17 × 10^2^	−3.81

**Table 3:**
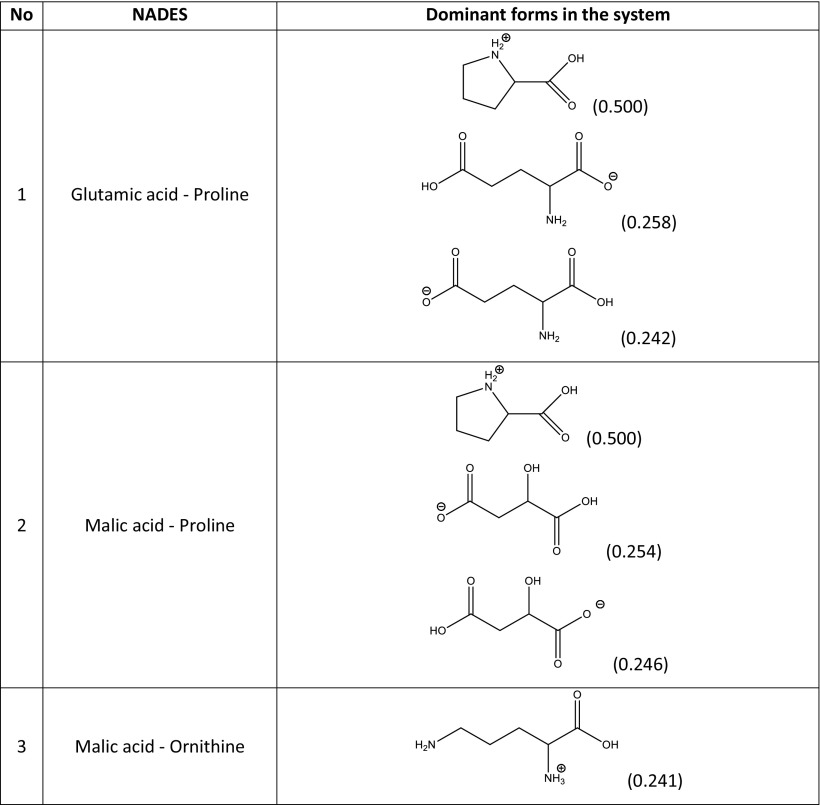

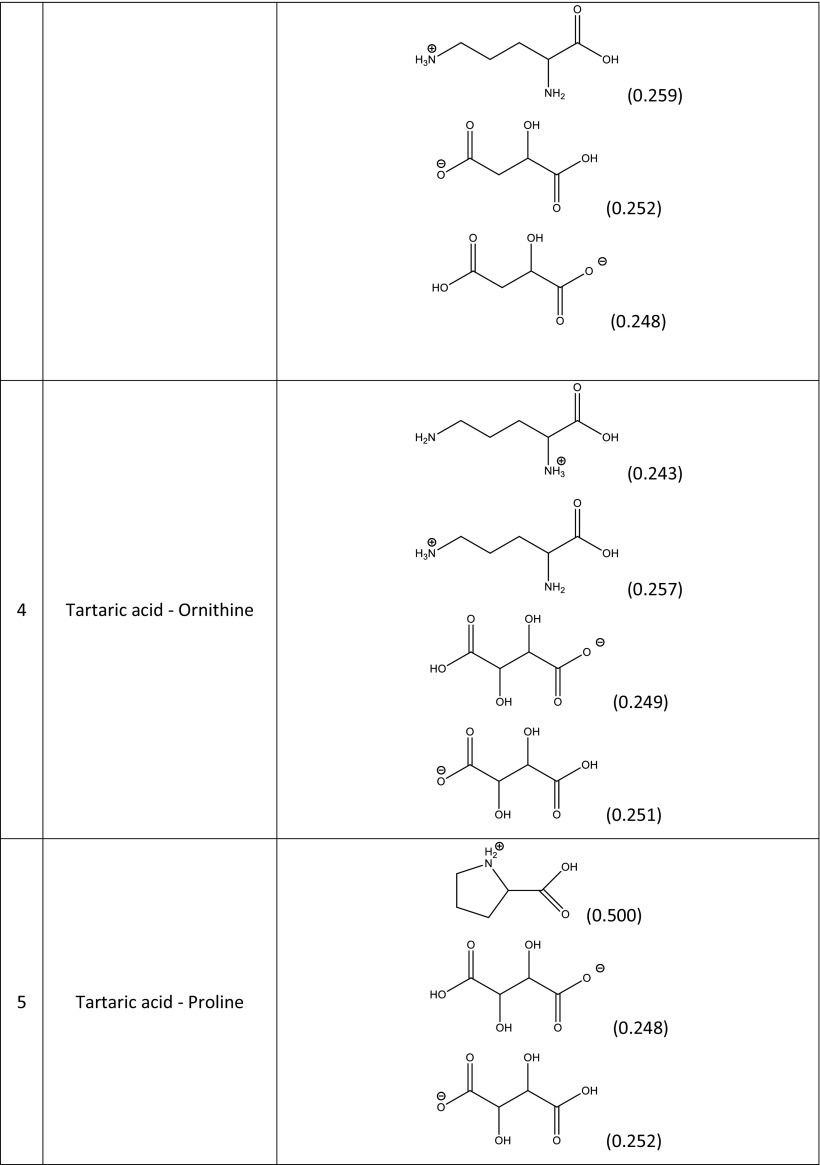

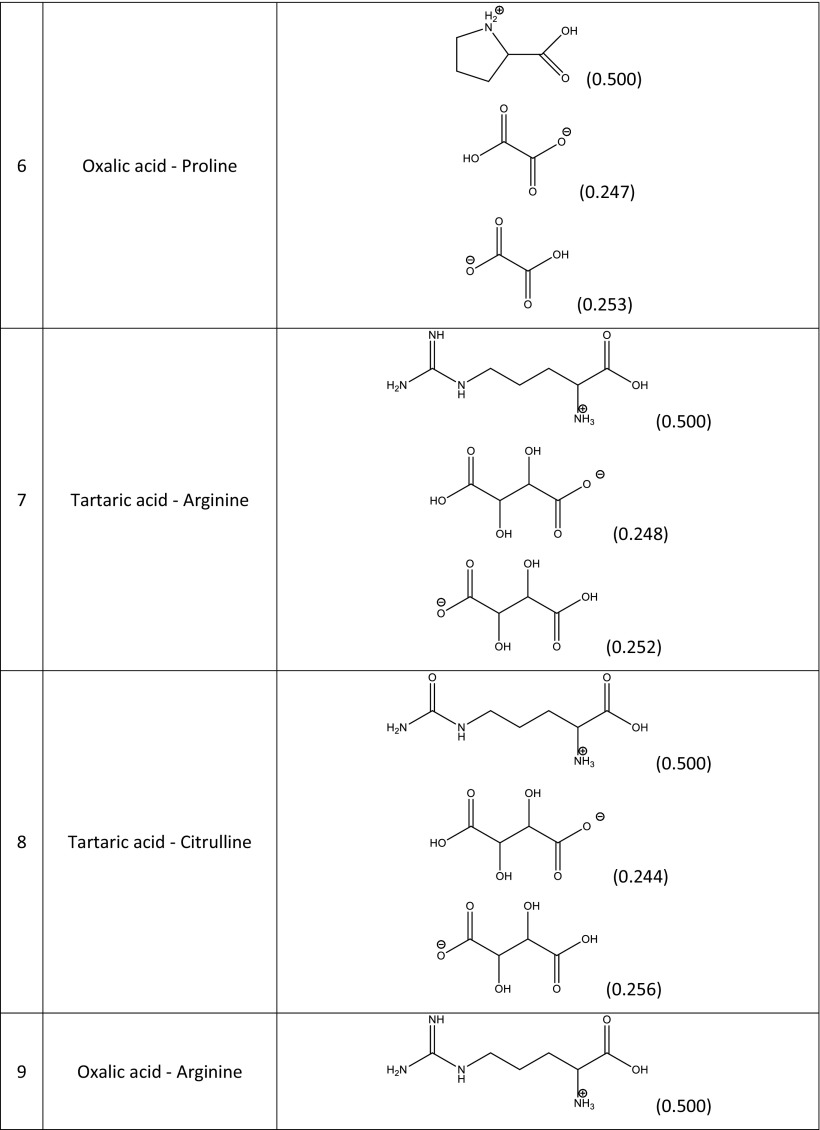

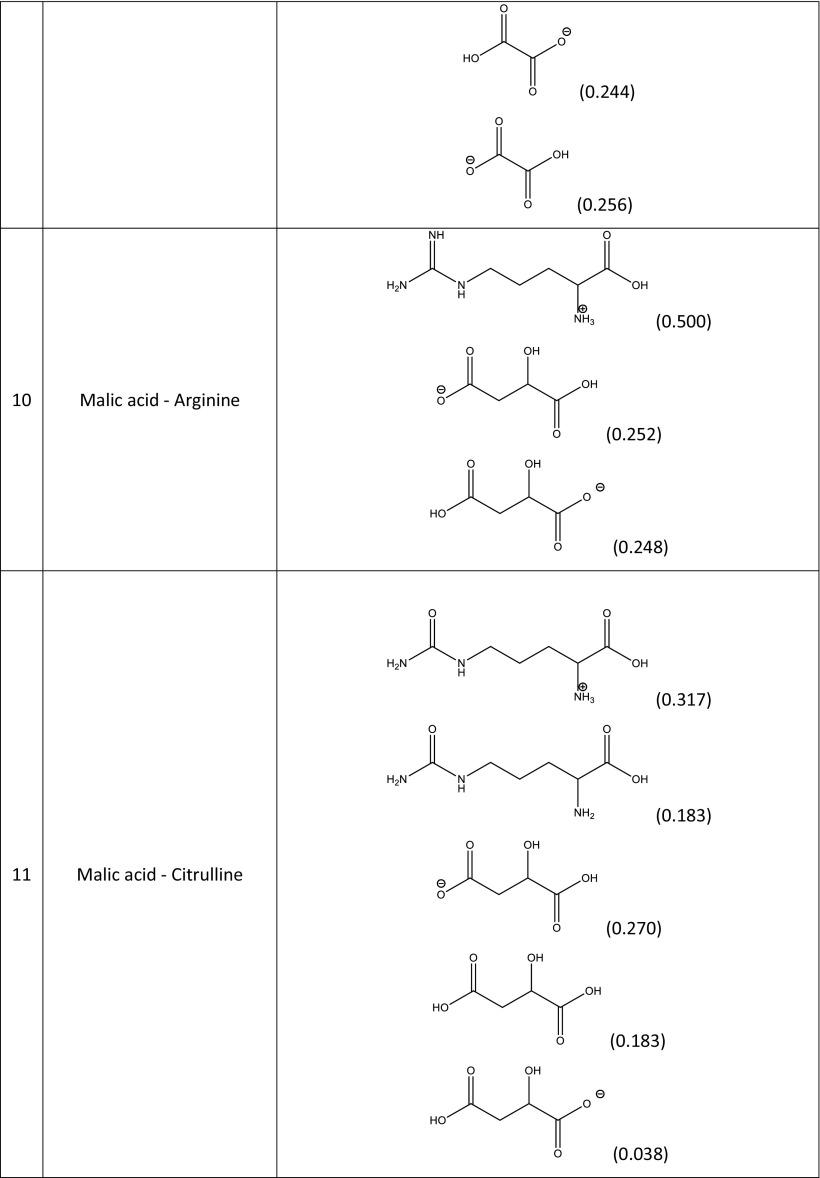

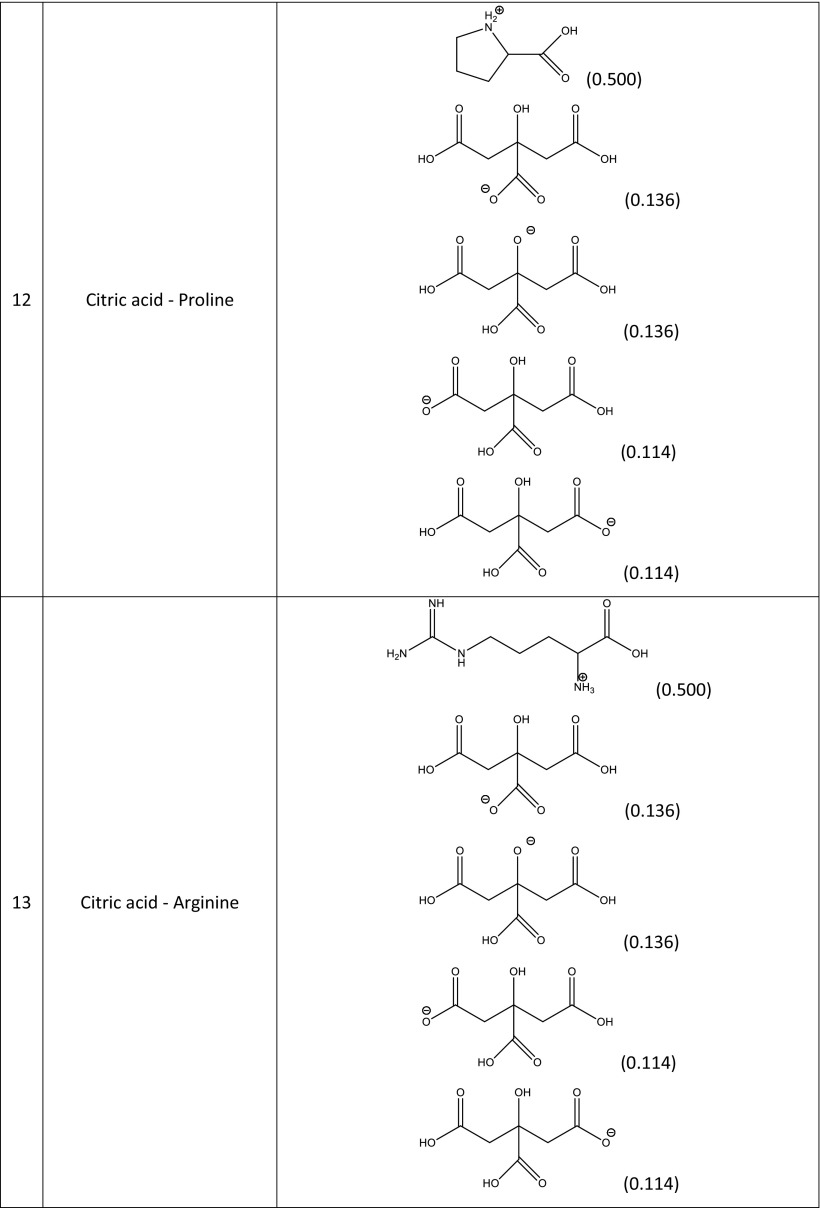

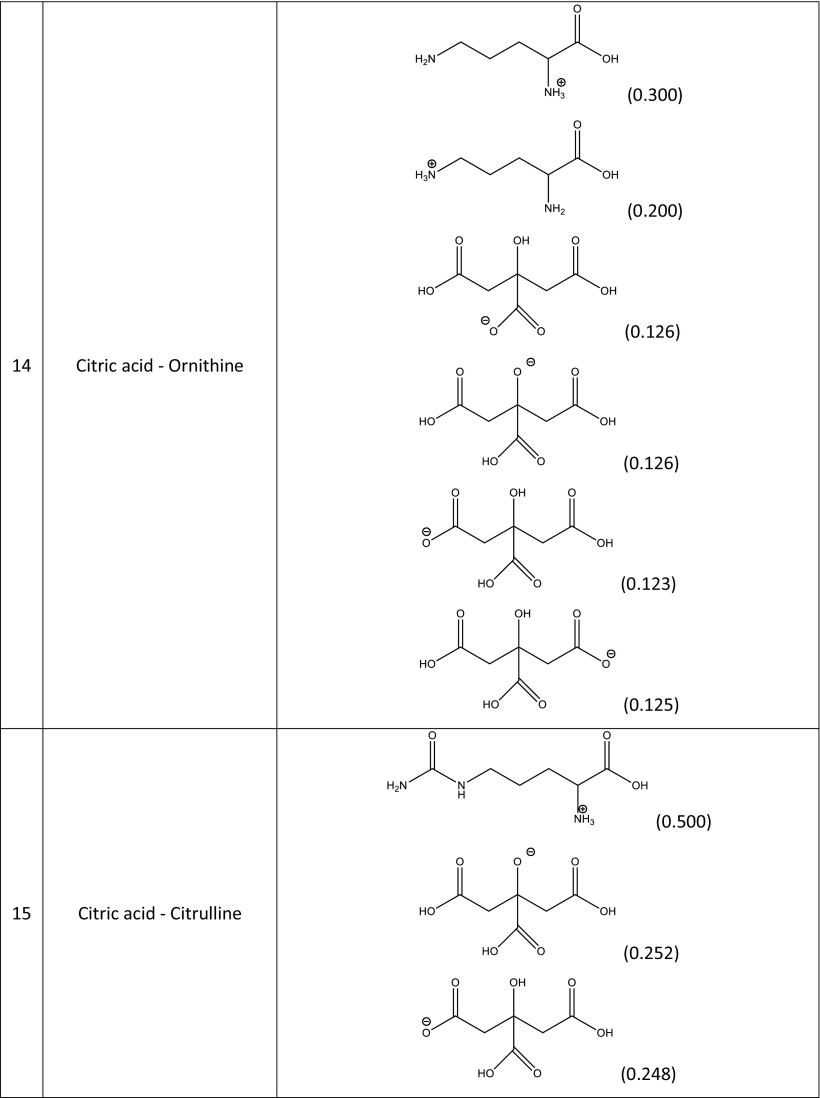
Dominant forms of the amino acids and organic acids in the natural deep eutectic solvents used to validate the constructed model

The resulting almost linear correlation of the solubility of rutin with its computed activity coefficient is a fortunate outcome that allows us to screen for other NADES with enhanced rutin dissolution capabilities. It also suggests that the proposed model of deep eutectic solvents is rational. It is worth mentioning that the largest discrepancies between the computed and predicted values were observed in the region of low rutin solubility, which is not very important because it is the high-solubility region that is of great interest to us. In fact, for the glutamic acid–proline system, which exhibited the highest rutin solubility (2.26 mg/mL), the difference between the experimental and computed values of solubility was only 28.6 μg/mL, corresponding to a relative difference of 1.3%.

### Screening for NADES that show enhanced rutin solubility

**Fig. 4:**
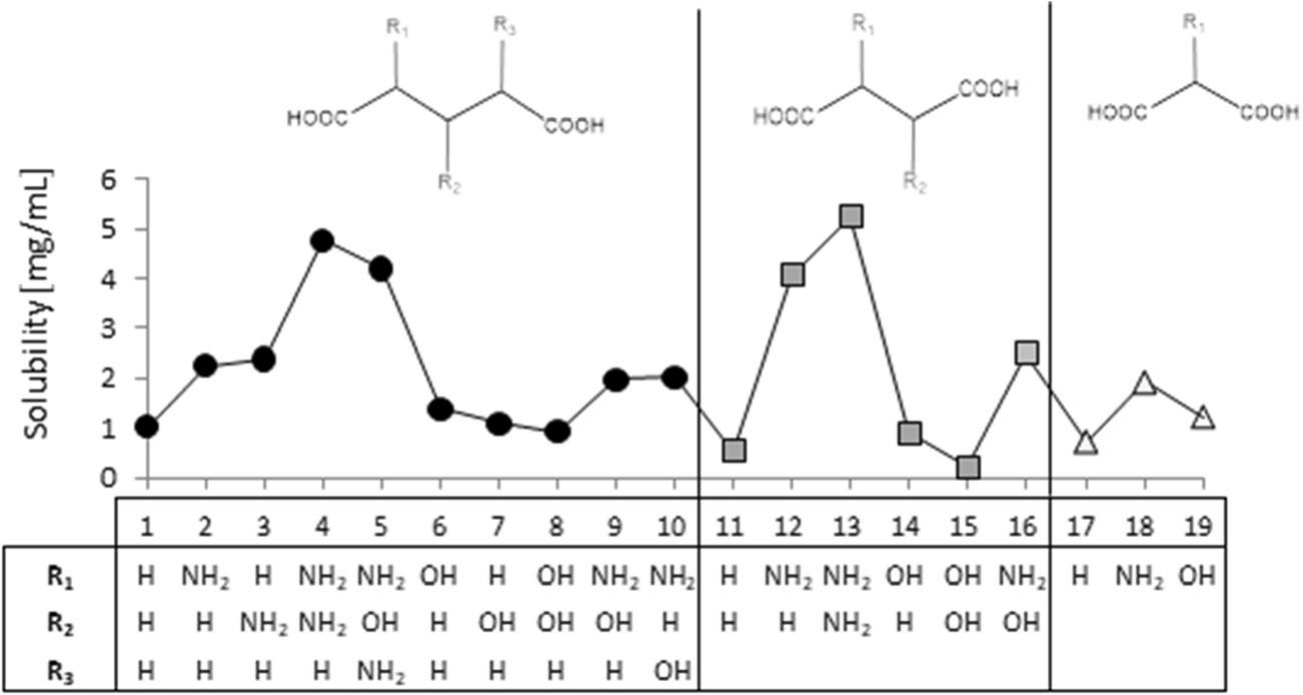
Calculated solubilities of rutin in deep eutectic solvents comprising proline and a selection of organic acids with two carboxyl groups. The numbers on the abscissa correspond to different functional group combinations in these acids, as defined in the table

A screening procedure was performed in order to identify natural deep eutectic solvents capable of solvating rutin more strongly than the solvents described above. For this purpose, 16 new carboxylic acids and two new amino acids were added to the pool of compounds used during the validation stage, which resulted in a total of 126 natural deep eutectic solvents. The procedure described in the section “Construction of the model and validation” was performed for all of these NADES; their compositions were determined by taking into account their dominant ionic forms and by performing computations of the corresponding reaction constants. The compositions of the best-performing systems are presented in Table 4. A comprehensive description of all the NADES considered in this work is provided in Table S1 of the ESM. The original set of NADES components was extended in a systematic manner, allowing for analyses of the relations between structure and performance in the new NADES. The studied carboxylic acids all had two carboxyl groups separated by an aliphatic chain of varying length that had hydroxyl or amino substituents. It was found that the number, position(s), and type(s) of functional groups present are crucial influences on the effectiveness of the studied NADES. Indeed, a wide range of values were seen for the rutin solubility estimated using the regression line provided in Fig. 3 and for the computed activity coefficient at infinite dilution, as shown in Table 5. The results for the new NADES showed that ten systems performed better than the best reference one. It is interesting to note that 2,3-diaminosuccinic acid was involved in four of these systems, and proline was involved in six of the systems. Indeed, the natural deep eutectic solvent comprising both of these compounds was found to be the best NADES for solvating rutin; its solubility was calculated to be as high as 5.25 mg/mL, representing a 130% increase in rutin dissolution compared to the proline–glutamic acid system. Two other NADES with significantly higher rutin solubilities than experimentally studied solvents were 2,4-diaminoglutaric acid—proline (4.78 mg/mL, 111% increase), 4-amino-3-hydroxyglutamic acid—proline (4.21 mg/ml, 86% increase), and aspartic acid–proline (4.06 mg/mL, 80% increase). It should also be noted that these NADES outperform water in terms of rutin solubility 34- to 44-fold (reference value: 120 μg/ml). Among the studied amino acids, those with a heterocyclic structure proved to be the most effective, as they were included in nine of the ten systems with the highest rutin solubilities. Optimizing the structure of the carboxylic acid to enhance the solubility of rutin involved varying the length of the main chain and the functional groups present in the acid. As shown in Fig. 4, which presents the solubilities of rutin in NADES comprising proline and different carboxylic acids, the highest solubilities (approximately 5.00 mg/mL) were obtained with carboxylic acids that had two amino groups attached to their chains. Substituting one of the NH_2_ groups for a hydroxyl group reduced the solubility of rutin, as did simply removing one of the amino groups, which resulted in a solubility of around 2.00 mg/mL. Carboxylic acids without any amino groups attached to the chain were characterized by even lower rutin solubilities. Those acids had either one or two hydroxyl groups as substituents or they had no substituents at all; in those cases, the solubility of rutin was around 1.00 mg/mL or less. As well as the substituents present, the length of the main chain of the carboxylic acid also influenced the solubility of rutin, since all of the acids studied had two carboxyl groups with some methylene groups between them. The highest solubility of rutin was obtained with acids that had a main chain consisting of two methylene groups.

**Table 4:**
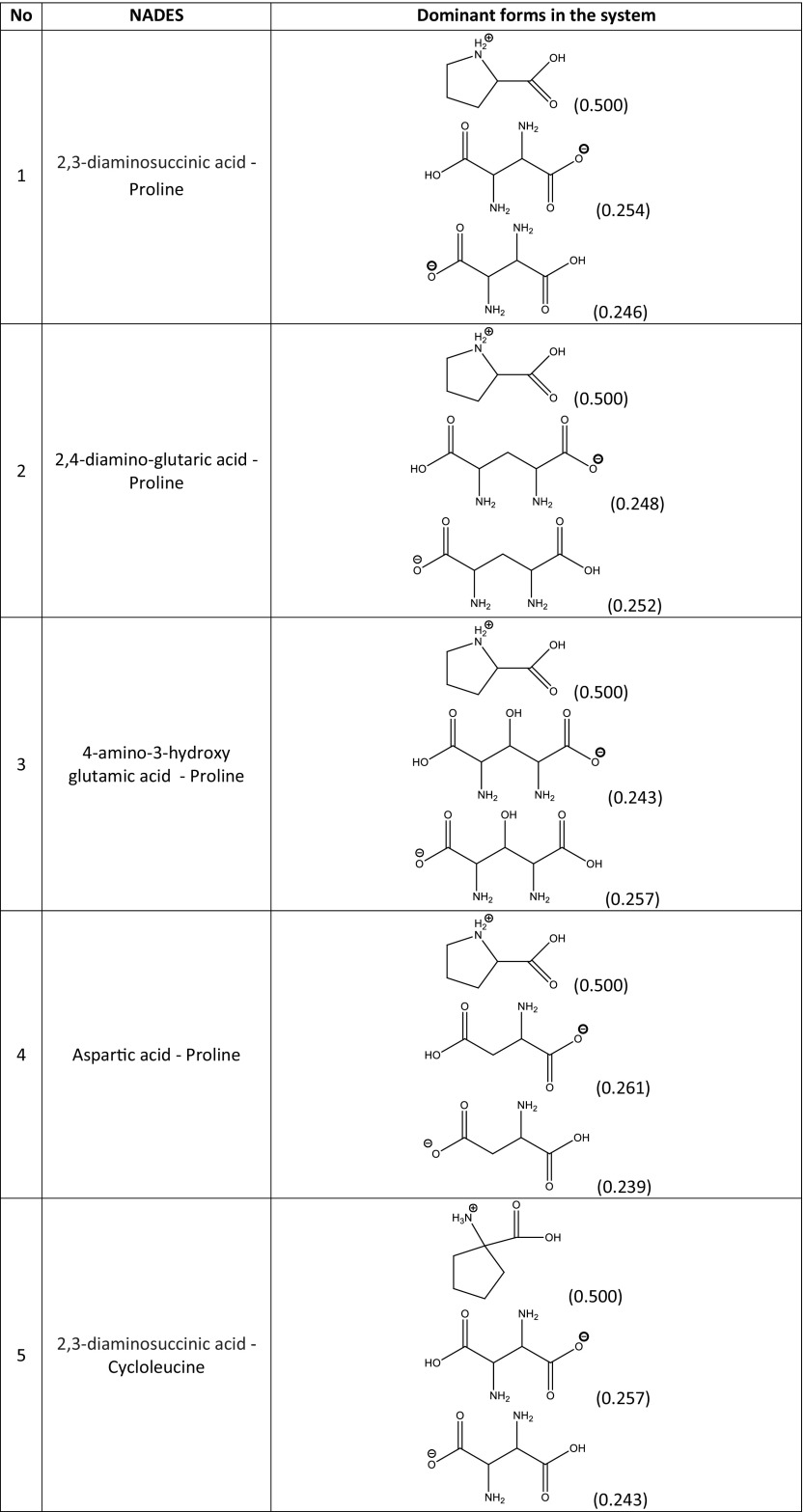

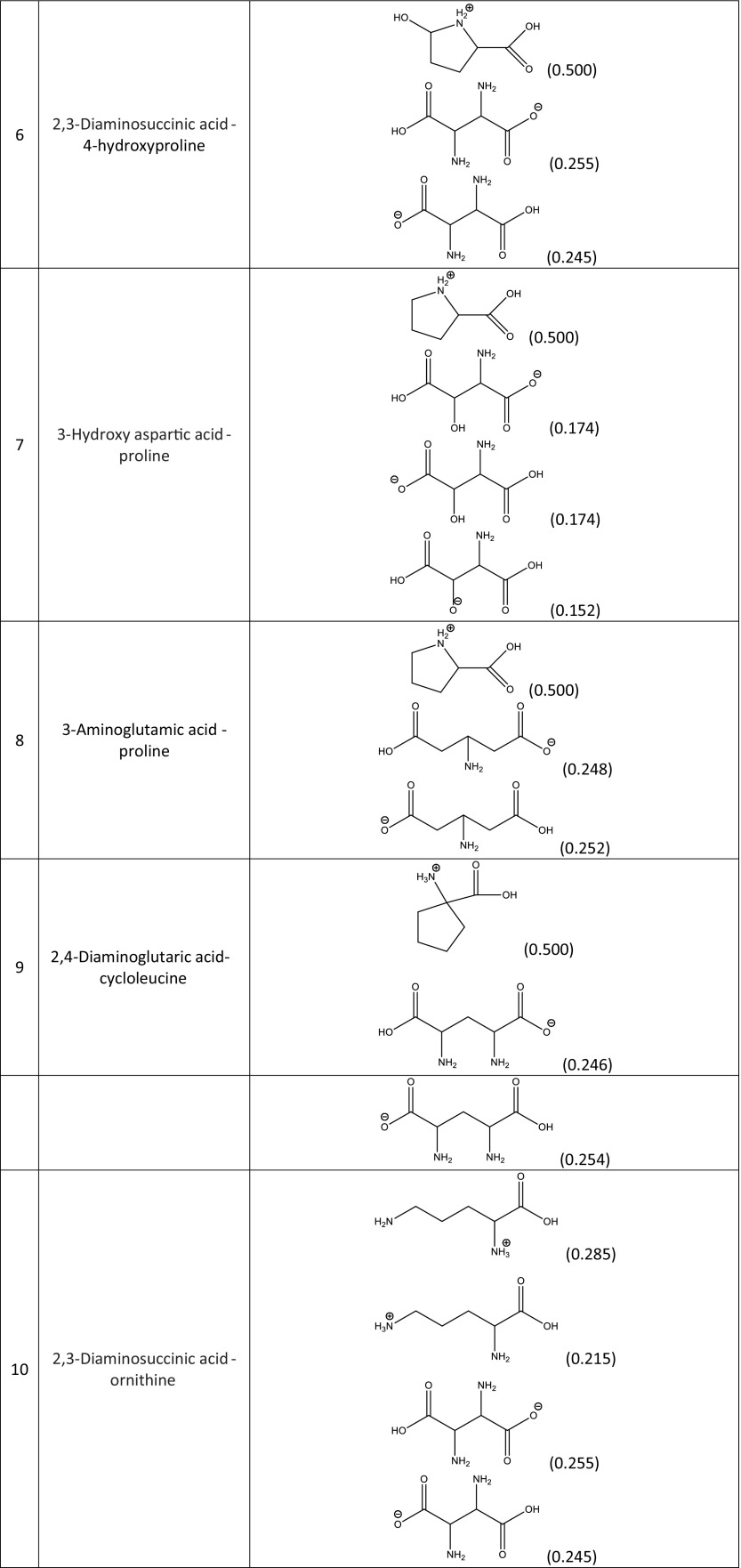
Dominant amino acid and organic acid forms in the deep eutectic solvents used when screening for solvents with particularly high rutin solubility

**Table 5:**
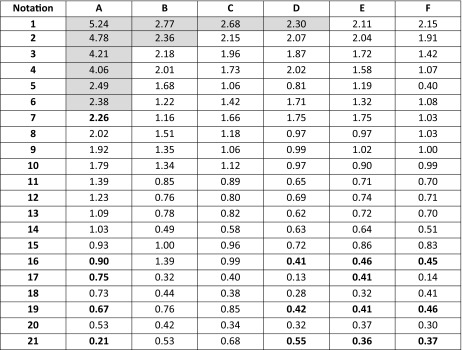
Solubilities of rutin (mg/mL) in various natural deep eutectic solvents

The experimental data were presented bold faced while others stand for estimated values. The values which exceed the experimental solubilities were highlighted. Notation of compounds is the same as in Table 1

**Fig. 5:**
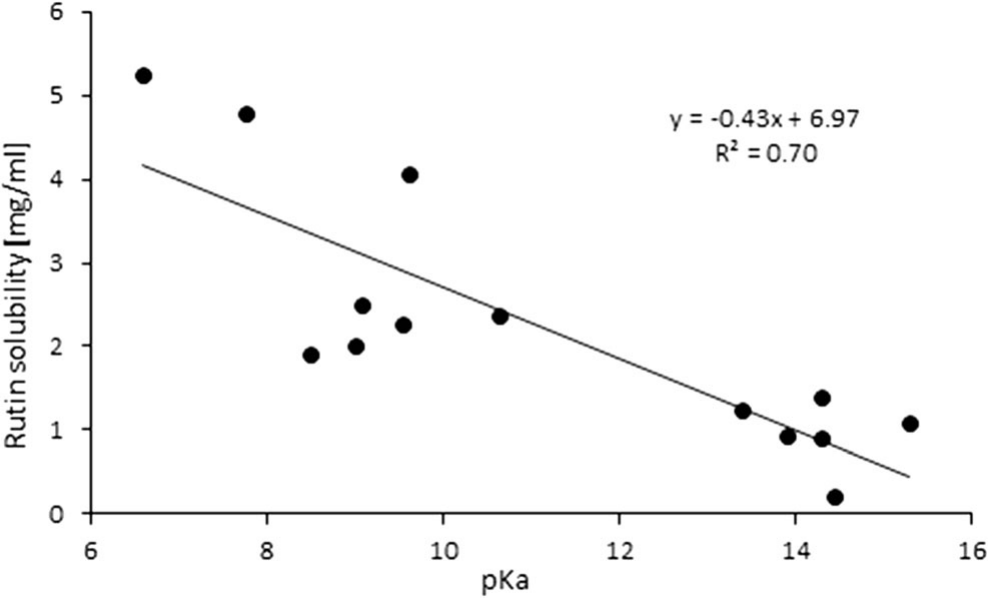
The relationship between predicted rutin solubility and the lowest p*K*_a_ values of substituents attached to the chain of the carboxylic acid. Detailed values are provided in the ESM (Table S2)

The high rutin-solvating power of carboxylic acids with attached amino groups is quite understandable. Rutin is a weak acid due to proton donation from phenolic groups attached to the aromatic ring, as illustrated in Fig. 1. According to ChemAxon [10], the lowest p*K*_a_ value for rutin is about 6.4. We would then expect that the presence of basic centers on the components of the solution mixture will help to promote direct heteromolecular contacts and enhance solubility. Of course, amino acids offer such basic sites. However, the acidic components in the considered NADES also contain amino groups, enhancing potential interactions with rutin. Additionally, the acidity of the carboxylic acid is an important aspect since it affects the dissociation of the basic centers of the amino acids. It is then reasonable to expect that these two aspects are important from the perspective of selecting the best NADES components for solvating rutin. Unfortunately, for substituted carboxylic acids, there are incongruent trends between the ability to deprotonate the carboxylic acid groups and the protonation potential of amino groups attached to the chain. This can be observed by inspecting the microacidities of the considered centers. Despite the fact that there are no relevant experimental data, estimated values can still be valuable in the above context. For this purpose, the ChemAxon [10] facilities were used to compute p*K*_a_ values, and the detailed results are collected in the ESM (see Table S2). Finally, qualitative trends can be inferred from these data. It is quite obvious that elongating the aliphatic chain reduces the acidity of the dicarboxylic acid and that adding hydroxyl or amino groups increases the acidity of the derivative. Furthermore, the amino substituents play a much more important role since they significantly increase the acidity of the substituted carboxylic acid. Also, amino groups behave differently depending on their environment. These two effects are cumulative but are not necessary additive. However, quite interesting trends that demonstrate the influence of the NADES composition on the solubility of rutin can be inferred. Indeed, there is a correlation between the acidity of the substituent attached to the carboxylic acid in a proline-containing NADES and the solubility of rutin in that NADES. Figure 5 shows the corresponding plot for this correlation, which implies that the more basic the substituent in the chain of the carboxylic acid, the greater the solubility of rutin. Thus, any further screening should take into account the abovementioned aspects, as the factors that enhance rutin solubility are high acidity of the carboxylic acid component of the NADES and a large number of highly basic centers. Also, the amino acid component of the NADES should have centers that are as basic as possible.

## Conclusions

The COSMO-RS methodology appears to be a useful tool for predicting the solubility of rutin in natural deep eutectic solvents, thus making it possible to screen for the most effective NADES to solvate rutin. This approach makes use of observed linear relationships between experimentally obtained data and computed values of the activity coefficient in the infinite dilution approximation. The resulting regression line, characterized by a coefficient of determination* R*^2^ of 0.974, allowed us to identify NADES with a 130% increase in rutin solubility compared to the reference solvent of rutin. This procedure does, however, require an accurate definition of the solvent. This is a crucial step when implementing theoretical modeling using NADES, as a trivial model that considered only neutral, undissociated species was found to be completely unrealistic. A more accurate model should account for all possible ionic forms that could occur in solution. The concentrations of these forms can be computed based on estimated values for the reaction constants of all chemically possible reactions. This general strategy can be applied to model any multicomponent system, including NADES, that has not been studied experimentally.

We screened a set of 126 NADES based on 21 carboxylic acids and 6 amino acids for solvents with enhanced rutin solubilities. Ten of these systems outperformed the reference system, which was characterized by a rutin solubility of 2.26 mg/mL. Among these systems, four proved to be particularly effective: 2,3-diaminosuccinic acid–proline (5.25 mg/mL), 2,4-diaminoglutaric acid–proline (4.78 mg/mL), 4-amino-3-hydroxyglutamic acid–proline (4.21 mg/ml), and aspartic acid–proline (4.06 mg/mL). The latter system seems to be particularly interesting, as it combines an 80% increase in rutin solubility with moderate cost of the chemicals required. A structural analysis led to the discovery of important traits in the structures of the best-performing systems. The amino acids that permitted the highest rutin solubilities all had a cyclic structure. The highest rutin solubility was achieved using carboxylic acids in which the main chain consisted of two methylene groups and had two amino groups attached as substituents. Because of the acidic properties of rutin, the presence of basic sites in the structures of the NADES components will generally enhance the solubility of rutin. This can be achieved by either enhancing the acidity of the carboxylic acid component, which in in turn promotes the dissociation of the amino acid component, or by providing strongly basic centers on the chain of the carboxylic acid, which promotes direct intermolecular contact with rutin.

## Electronic supplementary material

ESM 1 (DOCX 267 kb)
