# Supplementary material for: Application of a computational model of natural deep eutectic solvents utilizing the COSMO-RS approach for screening of solvents with high solubility of rutin
Source: J Mol Model. 2018 Jun 27;24(7):180. doi: 10.1007/s00894-018-3700-1 (PMC6021465; doi:10.1007/s00894-018-3700-1)
Supplement: Supplementary file 1 — (DOCX 267 kb) [file 894_2018_3700_MOESM1_ESM.docx]

**Supplementary Materials**

**Table S1** Dominant forms of amino acids and organic acids in the deep eutectic solvents used in screening for the best solubility of Rutin. The molar fractions given in parentheses were used for the computations of activity of Rutin in the mixture. The systems with lower solubility of Rutin than the best reference system were shown here

| **No** | **NADES** | **Dominant forms in the system** |
| --- | --- | --- |
| **1** | 2,3-diaminosuccinic acid - Arginine |  (0.500)   (0.259)   (0.241) |
| **2** | 2,3-diaminosuccinic acid - Citrulline |  (0.500)   (0.254)   (0.246) |
| **3** | 2,4-diamino-glutaric acid - 4-hydroxyproline |  (0.500)   (0.247)   (0.253) |
| **4** | 2,4-diamino-glutaric acid - Ornithine |  (0.275)   (0.225)   (0.246)   (0.254) |
| **5** | 2,4-diamino-glutaric acid - Arginine |  (0.500)   (0.246)   (0.254) |
| **6** | 2,4-diamino-glutaric acid - Citrulline |  (0.500)   (0.246)   (0.254) |
| **7** | Aspartic acid - Cycloleucine |  (0.500)   (0.250)   (0.250) |
| **8** | Aspartic acid - 4-Hydroxyproline |  (0.243)   (0.257)   (0.088)   (0.155)   (0.257) |
| **9** | Aspartic acid - Ornithine |  (0.261)   (0.239)   (0.253)   (0.247) |
| **10** | Aspartic acid - Arginine |  (0.500)   (0.251)   (0.249) |
| **11** | Aspartic acid - Citrulline |  (0.500)   (0.252)   (0.248) |
| **12** | 3-hydroxy aspartic acid - Cycloleucine |  (0.500)   (0.189)   (0.189)   (0.122) |
| **13** | 3-hydroxy aspartic acid - 4-Hydroxyproline |  (0.500)   (0.181)   (0.181)   (0.138) |
| **14** | 3-hydroxy aspartic acid - Ornithine |  (0.272)   (0.228)   (0.183)   (0.183)   (0.134) |
| **15** | 3-hydroxy aspartic acid - Arginine |  (0.500)   (0.177)   (0.177)   (0.146) |
| **16** | 3-hydroxy aspartic acid - Citrulline |  (0.500)   (0.172)   (0.172)   (0.154) |
| **17** | 3-Aminopentanedioic acid - Cycloleucine |  (0.500)   (0.246)   (0.254) |
| **18** | 3-Aminopentanedioic acid - 4-Hydroxyproline |  (0.500)   (0.249)   (0.251) |
| **19** | 3-Aminopentanedioic acid - Ornithine |  (0.285)   (0.215)   (0.249)   (0.251) |
| **20** | 3-Aminopentanedioic acid - Arginine |  (0.500)   (0.246)   (0.254) |
| **21** | 3-Aminopentanedioic acid - Citrulline |  (0.500)   (0.242)   (0.258) |
| **22** | Glutamic acid - Cycloleucine |  (0.500)   (0.245)   (0.255) |
| **23** | Glutamic acid - 4-hydroxyproline |  (0.500)   (0.247)   (0.253) |
| **24** | Glutamic acid - Ornithine |  (0.280)   (0.220)   (0.247)   (0.253) |
| **25** | Glutamic acid - Arginine |  (0.500)   (0.248)   (0.252) |
| **26** | Glutamic acid - Citrulline |  (0.500)   (0.249)   (0.251) |
| **27** | 2-amino-3-hydroxypentanedioic acid - Proline |  (0.500)   (0.203)   (0.203)   (0.094) |
| **28** | 2-amino-3-hydroxypentanedioic acid - Cycloleucine |  (0.500)   (0.201)   (0.201)   (0.098) |
| **29** | 2-amino-3-hydroxypentanedioic acid - 4-hydroxyproline |  (0.500)   (0.201)  (0.201)  (0.098) |
| **30** | 2-amino-3-hydroxypentanedioic acid - Ornithine | (0.277)  (0.223)  (0.197)  (0.197)  (0.106) |
| **31** | 2-amino-3-hydroxypentanedioic acid - Arginine | (0.500)  (0.200)  (0.200)  (0.100) |
| **32** | 2-amino-3-hydroxypentanedioic acid - Citrulline | (0.500)  (0.199)  (0.199)  (0.102) |
| **33** | Aminomalonic acid - Proline | (0.500)  (0.250)  (0.250) |
| **34** | Aminomalonic acid - Cycloleucine | (0.500)  (0.248)  (0.252) |
| **35** | Aminomalonic acid - 4-hydroxyproline | (0.500)  (0.249)  (0.251) |
| **36** | Aminomalonic acid - Ornithine | (0.276)  (0.224)  (0.249)  (0.251) |
| **37** | Aminomalonic acid - Arginine | (0.500)  (0.250)  (0.250) |
| **38** | Aminomalonic acid - Citrulline | (0.500)  (0.250)  (0.250) |
| **39** | 2-amino-4-hydroxypentanedioic acid - Proline | (0.500)  (0.207)  (0.207)  (0.086) |
| **40** | 2-amino-4-hydroxypentanedioic acid - Cycloleucine | (0.500)  (0.201)  (0.201)  (0.098) |
| **41** | 2-amino-4-hydroxypentanedioic acid - 4-Hydroxyproline | (0.500)  (0.189)  (0.189)  (0.122) |
| **42** | 2-amino-4-hydroxypentanedioic acid - Ornithine | (0.270)  (0.230)  (0.189)  (0.189)  (0.122) |
| **43** | 2-amino-4-hydroxypentanedioic acid - Arginine | (0.500)  (0.193)  (0.193)  (0.114) |
| **44** | 2-amino-4-hydroxypentanedioic acid - Citrulline | (0.500)  (0.191)  (0.191)  (0.118) |
| **45** | 2-hydroxypentanedioic acid - Proline | (0.500)  (0.198)  (0.198)  (0.104) |
| **46** | 2-hydroxypentanedioic acid - Cycloleucine | (0.500)  (0.197)  (0.197)  (0.106) |
| **47** | 2-hydroxypentanedioic acid - 4-hydroxyproline | (0.260)  (0.240)  (0.103)  (0.103)  (0.054)  (0.240) |
| **48** | 2-hydroxypentanedioic acid - Ornithine | (0.268)  (0.232)  (0.193)  (0.193)  (0.114) |
| **49** | 2-hydroxypentanedioic acid - Arginine | (0.500)  (0.199)  (0.199)  (0.102) |
| **50** | 2-hydroxypentanedioic acid - Citrulline | (0.297)  (0.203)  (0.115)  (0.115)  (0.067)  (0.203) |
| **51** | Hydroxymalonic acid - Proline | (0.500)  (0.203)  (0.203)  (0.094) |
| **52** | Hydroxymalonic acid - Cycloleucine | (0.500)  (0.200)  (0.200)  (0.100) |
| **53** | Hydroxymalonic acid - 4-hydroxyproline | (0.267)  (0.233)  (0.103)  (0.103)  (0.061)  (0.233) |
| **54** | Hydroxymalonic acid - Ornithine | (0.261)  (0.239)  (0.196)  (0.196)  (0.108) |
| **55** | Hydroxymalonic acid - Arginine | (0.500)  (0.193)  (0.193)  (0.114) |
| **56** | Hydroxymalonic acid - Citrulline | (0.281)  (0.219)  (0.112)  (0.112)  (0.057)  (0.219) |
| **57** | 3-hydroxypentanedioic acid - Proline | (0.500)  (0.206)  (0.206)  (0.088) |
| **58** | 3-hydroxypentanedioic acid - Cycloleucine | (0.500)  (0.203)  (0.203)  (0.094) |
| **59** | 3-hydroxypentanedioic acid - 4-hydroxyproline | (0.267)  (0.233)  (0.100)  (0.100)  (0.067)  (0.233) |
| **60** | 3-hydroxypentanedioic acid - Ornithine | (0.259)  (0.241)  (0.205)  (0.205)  (0.090) |
| **61** | 3-hydroxypentanedioic acid - Arginine | (0.500)  (0.204)  (0.204)  (0.092) |
| **62** | 3-hydroxypentanedioic acid - Citrulline | (0.281)  (0.219)  (0.109)  (0.109)  (0.063)  (0.219) |
| **63** | Glutaric acid - Proline | (0.500)  (0.253)  (0.247) |
| **64** | Glutaric acid - Cycloleucine | (0.500)  (0.252)  (0.248) |
| **65** | Glutaric acid - 4-hydroxyproline | (0.500)  (0.253)  (0.247) |
| **66** | Glutaric acid - Ornithine | (0.263)  (0.237)  (0.251)  (0.249) |
| **67** | Glutaric acid - Arginine | (0.500)  (0.251)  (0.249) |
| **68** | Glutaric acid - Citrulline | (0.262)  (0.238)  (0.131)  (0.131)  (0.238) |
| **69** | 2,4-dihydroxypentanedioic acid - Proline | (0.500)  (0.165)  (0.165)  (0.085)  (0.085) |
| **70** | 2,4-dihydroxypentanedioic acid - Cycloleucine | (0.500)  (0.160)  (0.160)  (0.090)  (0.090) |
| **71** | 2,4-dihydroxypentanedioic acid - 4-hydroxyproline | (0.272)  (0.228)  (0.091)  (0.091)  (0.045)  (0.045)  (0.228) |
| **72** | 2,4-dihydroxypentanedioic acid - Ornithine | (0.270)  (0.230)  (0.156)  (0.156)  (0.094)  (0.094) |
| **73** | 2,4-dihydroxypentanedioic acid - Arginine | (0.500)  (0.153)  (0.153)  (0.097)  (0.097) |
| **74** | 2,4-dihydroxypentanedioic acid - Citrulline | (0.272)  (0.228)  (0.082)  (0.082)  (0.054)  (0.054)  (0.228) |
| **75** | Malic acid - Cycloleucine | (0.500)  (0.252)  (0.248) |
| **76** | Malic acid - 4-hydroxyproline | (0.265)  (0.235)  (0.142)  (0.123)  (0.235) |
| **77** | Oxalic acid - Cycloleucine | (0.500)  (0.252)  (0.248) |
| **78** | Oxalic acid - 4-hydroxyproline | (0.258)  (0.242)  (0.128)  (0.130)  (0.242) |
| **79** | Oxalic acid - Ornithine | (0.278)  (0.222)  (0.253)  (0.247) |
| **80** | Oxalic acid - Citrulline | (0.276)  (0.224)  (0.136)  (0.140)  (0.224) |
| **81** | Malonic acid - Proline | (0.500)  (0.253)  (0.247) |
| **82** | Malonic acid - Cycloleucine | (0.500)  (0.254)  (0.246) |
| **83** | Malonic acid - 4-hydroxyproline | (0.255)  (0.245)  (0.129)  (0.125)  (0.245) |
| **84** | Malonic acid - Ornithine | (0.268)  (0.232)  (0.254)  (0.246) |
| **85** | Malonic acid - Arginine | (0.500)  (0.257)  (0.243) |
| **86** | Malonic acid - Citrulline | (0.265)  (0.235)  (0.135)  (0.130)  (0.235) |
| **87** | Citric acid - Cycloleucine | (0.500)  (0.252)  (0.248) |
| **88** | Citric acid - 4-hydroxyproline | (0.500)  (0.253)  (0.247) |
| **89** | Succinic acid - Proline | (0.500)  (0.255)  (0.245) |
| **90** | Succinic acid - Cycloleucine | (0.500)  (0.254)  (0.246) |
| **91** | Succinic acid - 4-hydroxyproline | (0.260)  (0.240)  (0.134)  (0.126)  (0.240) |
| **92** | Succinic acid - Ornithine | (0.259)  (0.241)  (0.254)  (0.246) |
| **93** | Succinic acid - Arginine | (0.500)  (0.257)  (0.243) |
| **94** | Succinic acid - Citrulline | (0.266)  (0.234)  (0.256)  (0.244) |
| **95** | Tartaric acid - Cylcoleucine | (0.500)  (0.244)  (0.256) |
| **96** | Tartaric acid - 4-hydroxyproline | (0.263)  (0.237)  (0.134)  (0.129)  (0.237) |
| **97** | 4-amino-3-hydroxy glutamic acid - Cycloleucine | (0.500)  (0.241)  (0.259) |
| **98** | 4-amino-3-hydroxy glutamic acid - 4-hydroxyproline | (0.500)  (0.242)  (0.258) |
| **99** | 4-amino-3-hydroxy glutamic acid - Arginine | (0.243)  (0.257) |
| **100** | 4-amino-3-hydroxy glutamic acid - Citrulline | (0.500)  (0.244)  (0.256) |
| **101** | 4-amino-3-hydroxy glutamic acid - Ornithine | (0.261)  (0.239)  (0.245)  (0.255) |

**Table S2** Estimated pKa values for the carboxylic acids used as NADES constituents

| **General formula** | **Carboxylic acids** | **Substituents** | | | **pKa values** | | |
| --- | --- | --- | --- | --- | --- | --- | --- |
|  |  | **R_1_** | **R_2_** | **R_3_** | **pKa_1_** | **pKa_2_** | **pKa_3_** |
|  | Glutaric acid | H | H | H | **-** | **-** | **-** |
|  | DL-Glutamic acid | NH_2_ | H | H | 9.54 | **-** | **-** |
|  | 2,4-diamino-glutaric acid | NH_2_ | NH_2_ | H | 9.49 | 7.75 | **-** |
|  | 3-Aminopentanedioic acid | H | NH_2_ | H | 10.64 | **-** | **-** |
|  | 2-hydroxypentanedioic acid | OH | H | H | 14.29 | **-** | **-** |
|  | 3-hydroxypentanedioic acid | H | OH | H | 15.31 | **-** | **-** |
|  | 2,4-dihydroxypentanedioic acid | OH | OH | H | 13.91 | 14.61 | **-** |
|  | 2-amino-3-hydroxypentanedioic acid | NH_2_ | OH | H | 9.00 | 14.46 | **-** |
|  | 2-amino-4-hydroxypentanedioic acid | NH_2_ | H | OH | 9.16 | **-** | 14.24 |
|  | 4-amino-3-hydroxy glutamic acid | NH_2_ | OH | NH_2_ | 7.21 | 14.05 | 8.96 |
|  | Succinic acid | H | H | - | **-** | **-** | **-** |
|  | Aspartic Acid | NH_2_ | H | - | 9.61 | **-** | **-** |
|  | 2,3-diaminosuccinic acid | NH_2_ | NH_2_ | - | 9.17 | 6.58 | **-** |
|  | 3-Hydroxy Aspartic Acid | NH_2_ | OH | - | 9.08 | 13.72 | **-** |
|  | DL-Malic acid | OH | H | - | 14.30 | **-** | **-** |
|  | DL-Tartaric acid | OH | OH | - | 14.44 | 13.03 | **-** |
|  | Malonic acid | H | - | - | **-** | - | - |
|  | Aminomalonic acid | NH_2_ | - | - | 8.50 | - | - |
|  | Hydroxymalonic acid | OH | - | - | 13.39 | - | - |
